# Effect of AI-driven simulation integrated with virtual case-based training on diagnostic and therapeutic reasoning homogenization among junior ophthalmologists: a prospective cohort study

**DOI:** 10.3389/fmed.2026.1812146

**Published:** 2026-09-18

**Authors:** Zhipeng Wu, Long Li

**Affiliations:** Department of Ophthalmology, The First Affiliated Hospital of Zhengzhou University, Zhengzhou, Henan, China

**Keywords:** artificial intelligence, clinical decision-making, diabetic retinopathy, diagnostic reasoning homogenization, simulation-based medical education, specialty training, virtual patient training

## Abstract

**Background:**

Conventional training models for diabetic retinopathy (DR) inadequately address the dynamic decision-making demands required for diagnostic reasoning standardization among junior ophthalmologists.

**Objective:**

To evaluate whether AI-driven simulation integrated with virtual DR case training is associated with greater homogenization of diagnostic and therapeutic reasoning compared with conventional case-based instruction.

**Methods:**

This single-center prospective cohort study enrolled 100 junior ophthalmologists (1–5 years’ clinical experience) assigned to an AI-integrated cohort (*n* = 50) or conventional cohort (*n* = 50). Both underwent 12-week, 48-hour curricula. Outcomes were assessed at baseline, post-intervention, and one-month post-intervention using blinded expert evaluation and algorithmic benchmarking.

**Results:**

Post-intervention, participants in the AI-integrated cohort showed significantly superior scheme-decision Kappa values (0.93 vs. 0.52; *p* < 0.001), key decision-point concordance (95.5% vs. 59.0%; *p* < 0.001), and differential diagnosis coverage (97.0% vs. 62.0%; *p* < 0.001). Diagnostic confidence (4.78 vs. 3.56; *p* < 0.001), complex-case diagnostic accuracy (94.0% vs. 60.0%; *p* < 0.001), and prognostic concordance with an independently validated reference model (RMSE: 0.31 vs. 1.52; *p* < 0.001) were markedly superior. Acute-complication response time was halved (11.4 vs. 21.3 min; *p* < 0.001), and high-risk-factor discrimination reached the high-discrimination classification (AUC: 0.95 vs. 0.72; *p* < 0.001). Individualized treatment-plan adaptation (90.4 ± 6.2 vs. 64.8 ± 7.4 on a 0–100 scale; *p* < 0.001) and comorbidity-association recognition (95.0% vs. 63.0%; *p* < 0.001) confirmed comprehensive practice competency gains. All advantages persisted at one-month follow-up (all *p* < 0.001), with cross-center consultation Kappa of 0.89 versus 0.49 and follow-up management accuracy of 92.0% versus 58.0%.

**Conclusion:**

AI-driven simulation integrated with virtual DR case training was associated with significantly enhanced reasoning homogenization, clinical confidence, and practical competency among junior ophthalmologists, suggesting that this integrated approach may offer a scalable, guideline-anchored model for standardized ophthalmic specialty training.

## 1 Introduction

Diabetic retinopathy (DR) remains the predominant microvascular complication of diabetes mellitus and constitutes a leading cause of preventable blindness worldwide. Global epidemiological data indicate a cumulative DR prevalence of approximately 34.6% among individuals with diabetes, while estimates from China place adult DR prevalence between 24.7% and 37.5%, with proliferative DR conferring a 25-fold elevated risk of blindness relative to the general population (1). The insidious progression of DR — characterized by subclinical early-stage pathology that may advance to sight-threatening complications including retinal neovascularization and vitreous hemorrhage — imposes a substantial burden on both patients’ quality of life and healthcare systems (2).

The accuracy and standardization of DR management are critically dependent on the clinical reasoning capabilities of frontline ophthalmologists. However, considerable heterogeneity persists in diagnostic and therapeutic proficiency among junior ophthalmologists across different hospital tiers and experience levels in China, with insufficient homogenization of clinical reasoning identified as a key bottleneck constraining improvements in national DR prevention and treatment outcomes (3). Consequently, the development of efficient, standardized training paradigms capable of systematically elevating the homogeneity of diagnostic reasoning among junior ophthalmologists specializing in DR carries significant clinical and public health implications.

Conventional training approaches for ophthalmologists have relied predominantly on case-based learning using real clinical cases, which, while effective in bridging theoretical knowledge with clinical practice, presents notable limitations. Traditional case presentations are inherently static, comprising fixed histories, imaging datasets, and outcomes that fail to capture the dynamic evolution of disease under therapeutic intervention and across individual patient variations (4). The emergence of virtual case-based simulation training has partially addressed these shortcomings by integrating multimodal ophthalmic imaging resources — including color fundus photography, fundus fluorescein angiography (FFA), optical coherence tomography (OCT), and OCT angiography (OCTA) — to construct high-fidelity diagnostic scenarios that strengthen lesion-recognition competencies across disease stages and complications (5). Nevertheless, this modality retains a predominantly retrospective feedback architecture, relying on *post-hoc* expert commentary rather than providing the immediate, targeted guidance necessary to correct diagnostic and therapeutic deviations in real time, thereby limiting its capacity to reinforce standardized clinical reasoning (6).

Advances in artificial intelligence (AI) simulation technology present a compelling opportunity to address these pedagogical gaps. AI-driven simulation platforms, grounded in deep learning architectures, incorporate intelligent feedback and evaluation modules that compare physicians’ diagnostic and treatment decisions against clinical practice guidelines and expert consensus standards in real time. These systems generate quantitative compliance scores, identify missed diagnoses and diagnostic errors, and deliver individualized optimization recommendations — enabling physicians to iteratively correct reasoning deviations and progressively consolidate standardized clinical thinking (7, 8). While existing evidence supports the independent efficacy of both virtual case-based simulation training in enhancing image recognition and case-analysis skills (5) and AI simulation in developing clinical decision-making competency (8), systematic investigation of their combined application for homogenization training of junior ophthalmologists remains absent from the literature.

The present study was therefore designed to address this evidence gap by constructing and evaluating an integrated pedagogical model combining AI-driven simulation with virtual DR case training. Through a prospective cohort design, we systematically assessed the association between this integrated approach and the homogenization of diagnostic and therapeutic decision-making, clinical decision-making confidence, depth of clinical reasoning, practical competency, and sustainability of training effects among junior ophthalmologists, with the aim of establishing a scientifically grounded, scalable pathway for standardized DR specialty training.

## 2 Materials and methods

### 2.1 Study design and participants

This single-center prospective cohort study was conducted at The First Affiliated Hospital of Zhengzhou University between June 2023 and June 2025. A total of 100 junior ophthalmologists undergoing structured specialty training were enrolled. In this study, “junior ophthalmologist” refers to a licensed ophthalmologist with 1–5 years of post-licensure clinical experience who is enrolled in a structured specialty training program, irrespective of their administrative professional title within the Chinese medical system (see Section 2.2 and Table 1 footnote for details on title classification). The study protocol was approved by the institutional ethics committee (approval number: FAU-ZU-121) and conducted in accordance with the principles of the Declaration of Helsinki. All participants received comprehensive information regarding the study objectives, intervention content, potential risks and benefits, and scope of data utilization, and provided written informed consent prior to enrollment. Participants retained the unconditional right to withdraw at any stage without impact on their professional development. All research data underwent de-identification processing and were stored using password-protected mechanisms, accessible solely for the academic analyses of this study.

**Table 1:** Baseline characteristics of the study cohorts.

Characteristic	AI-integrated cohort (*n* = 50)	Conventional cohort (*n* = 50)	Test statistic	*p*
Age (years), mean ± SD	36.24 ± 5.67	35.89 ± 5.83	t-test: df = 98; *p* = 0.756	0.756
Sex, *n* (%)			*χ*^2^ test: df = 1; *p* = 0.689	0.689
Male	30 (60.0)	28 (56.0)		
Female	20 (40.0)	22 (44.0)		
Professional title^a^, *n* (%)			*χ*^2^ test: df = 2; *p* = 0.845	0.845
Resident physician	27 (54.0)	25 (50.0)		
Attending physician	17 (34.0)	19 (38.0)		
Senior attending physician	6 (12.0)	6 (12.0)		
Prior DR training experience, *n* (%)	22 (44.0)	19 (38.0)	*χ*^2^ test: df = 1; *p* = 0.539	0.539
Independent DR case volume, *n* (%)			*χ*^2^ test: df = 2; *p* = 0.868	0.868
<30 cases	8 (16.0)	10 (20.0)		
30–60 cases	29 (58.0)	27 (54.0)		
>60 cases	13 (26.0)	13 (26.0)		
DR theoretical knowledge score, mean ± SD	78.45 ± 6.33	77.89 ± 6.58	t-test: df = 98; *p* = 0.660	0.660
Master's degree or higher, *n* (%)	48 (96.0)	47 (94.0)	Fisher's exact; *p* = 1.000	1.000
Annual DR image interpretations, *n* (%)			*χ*^2^ test: df = 2; *p* = 0.786	0.786
<500	16 (32.0)	18 (36.0)		
500–1000	23 (46.0)	23 (46.0)		
>1000	11 (22.0)	9 (18.0)		

DR, diabetic retinopathy; SD, standard deviation.

a Professional titles reflect the Chinese medical administrative classification system (*zhùyuàn yīshī*, *zhŭzhì yīshī*, and *fù zhŭrèn yīshī*, respectively) and are determined primarily by years of post-licensure service and national qualification examination performance. All participants met the inclusion criterion of 1–5 years’ clinical experience; title distribution does not indicate functional seniority equivalent to Western training-stage designations. See Section 2.2 for details.

To address the potential influence of real-world clinical exposure on outcome measurements, additional structural safeguards were embedded within the cohort framework. All enrolled physicians were undergoing structured specialty training within the general ophthalmology rotation program of the same institution and, throughout the 12-week intervention and the 4-week post-intervention follow-up, were assigned to comparable subspecialty rotations under uniform institutional scheduling policies. Concurrent clinical responsibilities—including outpatient consultations, inpatient ward duties, on-call emergency coverage, and assisted surgical participation—were rotated on a standardized calendar under the direct supervision of attending or associate chief physicians, without independent operative responsibility. Weekly clinical workload was tracked using institutional duty-roster records and was comparable between cohorts (mean 42.6 versus 42.1 h; *p* = 0.671). Self-directed learning activities, external continuing medical education participation, and preparation for professional advancement examinations were self-reported at biweekly intervals and did not differ significantly between the two cohorts (all *p* > 0.05). Balance of these clinical exposure variables is summarized in Supplementary Table S5.

### 2.2 Eligibility criteria

**Inclusion criteria:** (1) Licensed medical practitioner with 1–5 years of clinical ophthalmology experience enrolled in a structured specialty training program; (2) no prior exposure to formal DR-specific standardized training or AI simulation-based instruction; (3) voluntary participation with signed informed consent; (4) ability to complete the full 12-week intervention and all scheduled assessments.

**Exclusion criteria:** (1) Visiting or postgraduate fellowship physicians; (2) prior participation in comparable DR training research; (3) cognitive impairment precluding cooperation with assessment procedures; (4) withdrawal attributable to institutional transfer, resignation, or other administrative reasons.

**Note on professional titles:** In the Chinese medical system, professional titles reflect an administrative classification determined chiefly by years of post-licensure service and qualification examinations, and therefore do not correspond directly to Western residency or fellowship stages (20–23). All participants in the present study met the pre-specified inclusion criterion of 1–5 years of clinical experience irrespective of title; the title distribution shown in Table 1 is reported solely for demographic transparency and does not indicate functional seniority. A detailed exposition of the title system and its relationship to clinical competency certification is provided in Supplementary Note S1.

### 2.3 Cohort assignment and bias mitigation

Participants were assigned to one of two training cohorts based on their enrollment sequence in the specialty training program. Physicians enrolled in the AI-enhanced training track constituted the AI-integrated cohort (*n* = 50), while those enrolled in the standard training track formed the conventional cohort (*n* = 50). Given the non-randomized design, multiple strategies were implemented to minimize selection and measurement bias. First, baseline equivalence was verified across 10 demographic and professional variables (Table 1; all *p* > 0.05). Second, all outcome assessors, expert scoring panelists, and statistical analysts remained strictly blinded to cohort assignment throughout the study. Third, identical case materials, teaching faculty calibration, and evaluation instruments were employed across both cohorts to control for information bias. Fourth, propensity score analysis was conducted as a sensitivity analysis to assess the robustness of primary findings to potential residual confounding (see Supplementary Table S1).

### 2.4 Sample size estimation

Based on preliminary data, the expected post-intervention scheme-decision Kappa value was 0.52 ± 0.08 for the conventional cohort and 0.75 ± 0.06 for the AI-integrated cohort. With a two-sided significance level of *α* = 0.05, statistical power of 0.80 (*β* = 0.20), and an allocation ratio of 1:1, sample size calculations performed using PASS 15.0 yielded a minimum requirement of 45 participants per cohort (total *n* = 90). Accounting for an anticipated 10% attrition rate, 50 participants were enrolled per cohort, yielding a total sample of 100.

### 2.5 Interventions

Both cohorts underwent a 12-week structured curriculum comprising 48 contact hours to ensure equivalent training intensity. A detailed comparison of training components across cohorts is summarized in Supplementary Table S2.

#### 2.5.1 AI-integrated cohort

The AI-integrated training employed a deep learning–based DR diagnostic simulation platform comprising three core functional modules:

- **Multimodal Imaging Database.** The platform integrates four imaging modalities — color fundus photography, FFA, OCT, and OCTA — curated with strict reference to the Chinese Diabetic Retinopathy Clinical Practice Guidelines (2023 Edition). The database encompasses DR stages 1 through 5 and includes 200 de-identified clinical cases, of which 30% represent complex presentations with concurrent macular edema, glaucoma, cataract, or other comorbid conditions.
  **Model architecture and validation:** The image-analysis backbone employed a ResNet-50 convolutional neural network fine-tuned for DR staging and lesion segmentation. The model was trained internally on 12,480 de-identified fundus and OCT images collected from the institutional retinal-imaging archive, augmented with 4,200 publicly available images from the Kaggle EyePACS and Messidor-2 datasets. Two board-certified retinal specialists independently annotated each image using the Chinese Diabetic Retinopathy Clinical Practice Guidelines (2023 Edition) staging schema, with discrepancies resolved by a third senior adjudicator; inter-annotator agreement reached Cohen's *κ* = 0.91. The corpus was partitioned into training, validation, and test sets in a 70:15:15 ratio at the patient level to avoid image leakage, and external validation was performed on an independent 1,050-image test set from a collaborating tertiary center, yielding an area under the receiver operating characteristic curve of 0.94 for referable DR detection. To preclude cross-contamination between cohorts, access to the AI simulation platform was gated by individual institutional credentials, with authentication and usage logs restricted to the pre-specified curricular hours. Participants in the conventional cohort were not permitted platform access at any point during the study period.
- **Dynamic Disease-Evolution Engine.** This module simulates clinical trajectories including glycemic fluctuations, lesion progression or regression following therapeutic intervention, and acute complication triggers (vitreous hemorrhage, retinal detachment, neovascular glaucoma), supporting more than 10 branching disease pathways. The simulation logic is derived from longitudinal real-world clinical follow-up data and guideline-recommended management pathways. Each simulated trajectory integrates structured narrative text (history, laboratory results, clinical decision prompts) with authentic clinical images—including serial color fundus photographs, FFA sequences, and OCT scans—retrieved directly from the de-identified institutional archive rather than synthesized by generative artificial intelligence; consequently, no AI-generated imagery was presented to participants at any stage of training. A total of 60 dynamic case scenarios drawn from the 200-case master library were used in Phase II, spanning DR stages 2 through 5 with balanced representation of complicated and uncomplicated presentations.
- **Intelligent Feedback and Evaluation Module.** This module integrates a DR clinical practice guideline rule base with a consensus model developed by three senior DR subspecialists (each with ≥10 years of clinical experience). It provides real-time output of decision-compliance scores, missed-diagnosis and misdiagnosis alerts, and individualized optimization recommendations. Architecturally, this module is a hybrid system rather than a single monolithic model. Guideline compliance and decision-tree adjudication are executed by a rule-based clinical reasoning engine encoding the branching logic of the Chinese Diabetic Retinopathy Clinical Practice Guidelines (2023 Edition) and the expert-consensus algorithm; image-based staging and lesion identification are performed by the deep-learning classifier described in Section 2.5.1(a); and free-text physician entries (differential diagnoses, treatment rationale) are parsed by a domain-adapted natural-language-processing pipeline (based on a fine-tuned BioBERT model) that maps clinical terminology to standardized concept identifiers before rule-based scoring. Together, these components produce the compliance scores, missed-diagnosis alerts, and individualized recommendations delivered to the physician.

Training implementation followed a three-phase progressive structure:

**Phase I — Foundational Module (Weeks 1–4; 32 h):** DR clinical practice guideline instruction (4 h) and single-modality image recognition training (28 h). Guideline instruction, delivered by senior physicians who contributed to the expert-consensus model, covered DR staging criteria, key lesion-feature recognition, and fundamental treatment principles. During image-recognition training, the AI system calibrated case difficulty to each physician's proficiency level via pre-assessment, delivered real-time annotation of core lesion features (microaneurysms, hard exudates, cotton-wool spots), and provided dual-verification feedback (AI algorithm and expert consensus) following each staging diagnosis.

**Phase II — Advanced Module (Weeks 5–8; 8 h):** Dynamic case simulation training (2 h/week) in which physicians independently formulated investigation plans (e.g., the need for supplementary OCTA) and treatment strategies (pharmacotherapy, laser photocoagulation, vitrectomy). The system triggered corresponding disease-trajectory evolution in real time based on physician decisions and generated visual decision-pathway diagrams with guideline-concordance analysis reports.

**Phase III — Comprehensive Module (Weeks 9–12; 8 h):** Complex case training (2 h/week) using DR cases with interdisciplinary comorbidities (diabetic nephropathy, hypertension, coronary artery disease). Physicians completed the full diagnostic and therapeutic workflow — including differential diagnosis, individualized treatment planning, and acute-complication emergency management. Post-session, the system generated multidimensional evaluation reports encompassing key decision-point concordance and differential-diagnosis coverage metrics.

**Feedback mechanism:** Following each training session, the platform generated a structured Diagnostic and Therapeutic Decision Optimization Report containing: (1) deviation analysis referencing clinical practice guidelines, expert consensus, and evidence-based rationale; (2) an inventory of overlooked high-risk factors (e.g., suboptimal glycemic control, smoking history, hypertension); (3) a quantitative treatment-plan suitability score (0–100, incorporating lesion-stage matching, comorbidity consideration, patient compliance, risk–benefit ratio, and guideline concordance); and (4) targeted case recommendations to reinforce identified competency gaps.

#### 2.5.2 Conventional cohort

The conventional cohort received traditional case-based learning combined with virtual DR case training:

**Phase I — Didactic Lectures (Weeks 1–4; 32 h):** Delivered by the same panel of senior physicians contributing to the AI-integrated cohort's expert-consensus database, ensuring content equivalence. Curriculum comprised DR clinical practice guidelines (4 h) and image interpretation lectures (28 h, 7 h/week). Instruction utilized a blended format of in-person centralized teaching with online-replay review, and distributed static imaging materials and guideline reference documents identical in provenance to those of the AI-integrated cohort.

**Phase II — Case-Based Discussion (Weeks 5–12; 16 h):** Five static DR cases per week (matched to the AI-integrated cohort's baseline case characteristics; complete imaging data, de-identified medical record summaries) were distributed using a combined format of in-person small-group discussion (5 physicians/group) and online treatment-plan submission. Weekly expert-led review sessions (2 h) provided structured commentary on the strengths and limitations of submitted plans with general improvement recommendations. No real-time feedback or dynamic disease simulation was provided.

Virtual DR case training content was identical across both cohorts.

#### 2.5.3 Quality assurance

All clinical records used across both cohorts were identical and de-identified to protect patient privacy. All teaching and discussion faculty underwent standardized calibration training to delineate teaching content boundaries and feedback standards, minimizing information bias attributable to instructor variability. The complete teaching process was documented to verify content consistency and integrity across cohorts. Training session attendance was recorded for both cohorts, and a minimum completion threshold of ≥90% of scheduled sessions was required for inclusion in the per-protocol analysis.

### 2.6 Outcome measures

All outcomes were assessed at three pre-specified time points: baseline (T0, prior to the intervention), immediately post-intervention (T1, week 12), and one-month post-intervention (T2, week 16). All primary and secondary outcomes were assessed at T0 and T1. The three primary diagnostic/therapeutic decision-homogenization outcomes and the three sustainability indicators [Section 2.6.2(D)] were additionally assessed at T2.

**Independence of assessment from training tools:** To prevent measurement bias arising from familiarity with the training platform, all outcome scoring was performed independently of the AI simulation system used for instruction. The gold-standard references for scheme-decision Kappa, key decision-point concordance, differential diagnosis coverage, complex-case diagnostic accuracy, treatment-plan adaptation, and cross-center consultation consistency were derived from expert-consensus panels of three or more chief-physician-grade retinal subspecialists whose scoring rubrics were developed *a priori* and were not visible to participants during training. Expert scorers were blinded to cohort allocation and used standardized paper-based rubrics rather than the training platform's feedback interface. Where quantitative AI benchmarking was employed (namely for the prognostic-evaluation RMSE and the high-risk-factor early-warning AUC), the reference model was a separate research-only predictive algorithm trained on an independent longitudinal follow-up dataset and was not exposed to either cohort during the intervention. This separation between instructional and evaluative infrastructures ensures that between-group differences reflect clinical reasoning rather than shared familiarity with any single scoring engine.

#### 2.6.1 Primary outcome: diagnostic and therapeutic decision homogenization

- **Scheme-Decision Kappa Value.** The Kappa consistency coefficient was calculated between each physician's management plan and a gold-standard reference comprising the Chinese Diabetic Retinopathy Clinical Practice Guidelines (2023 Edition) and a consensus plan jointly formulated by three senior DR subspecialists (all holding chief-physician titles with ≥10 years of clinical experience). Interpretation thresholds: 0.00–0.20 (poor), 0.21–0.40 (fair), 0.41–0.60 (moderate), 0.61–0.80 (substantial), 0.81–1.00 (almost perfect agreement).
- **Key Decision-Point Concordance Rate (%).** Five core decision points were pre-specified: lesion-staging determination, macular edema assessment, initial treatment modality selection (pharmacotherapy/laser/surgery), follow-up frequency, and acute-complication early-warning activation. At each of these decision points, concordance with the reference standard was scored on a three-level ordinal rubric rather than as a strict binary indicator, in order to accommodate clinically meaningful intermediate cases such as those in which a physician selected the correct treatment modality but with suboptimal parameters or timing. The graded scoring assigned a value of 1.0 for full concordance with the guideline-plus-expert-consensus reference, 0.5 for partial concordance, and 0.0 for full discordance, with the criteria for each level pre-specified in the scoring rubric distributed to the blinded expert panel. The per-case concordance score was calculated as the sum of the five decision-point scores divided by 5, expressed as a percentage; per-case scores therefore fell on an 11-value grid ranging from 0% to 100% in 10-percentage-point increments. The per-participant concordance rate reported throughout the manuscript represents the arithmetic mean of the case-level concordance percentages computed across the standardized assessment battery of 10 diagnostic and therapeutic cases administered to every participant at each measurement time point (T0, T1, and T2, where applicable). Because per-case scores are drawn from an 11-value ladder (0%, 10%, 20%, and so on up to 100%), the mean across 10 cases yields a per-participant percentage on a 1-percentage-point grid (0%, 1%, 2%, and so on up to 100%), thereby producing a genuinely continuous outcome rather than the coarse 20% increments that would arise from strictly binary decision-point scoring. Group-level medians and interquartile ranges were then computed from these per-participant values using the R Type 7 quantile-interpolation algorithm (also the SPSS default), which linearly interpolates between neighboring ordered values and therefore produces reported summary statistics at a finer granularity than the 1% per-participant grid, accounting for values such as a median of 95.5% or an interquartile boundary of 93.8%. A fully worked example illustrating this calculation for a representative participant is provided in Supplementary Note S2, and the per-participant values underlying every summary statistic reported in Table 2 have been independently re-verified against the raw scoring sheets by two investigators.
- **Differential Diagnosis Coverage Rate (%).** Eight common differential diagnoses were pre-specified: age-related macular degeneration, retinal vein occlusion, central serous chorioretinopathy, uveitis, hypertensive retinopathy, retinal detachment, optic neuropathy, and congenital retinal dystrophy. Consideration of each of the eight differential entities was scored on the same three-level ordinal rubric used for the key decision-point metric, with 1.0 awarded when the differential was correctly identified together with an appropriate clinical justification, 0.5 when the differential was named without sufficient justification or listed with partially correct reasoning, and 0.0 when the differential was omitted or incorrectly considered. The per-case coverage score was calculated as the sum of the eight differential-level scores divided by 8, expressed as a percentage. Consistent with the aggregation strategy applied to the key decision-point concordance metric, each participant's differential diagnosis coverage rate was computed as the mean of case-level coverage percentages across the same battery of 10 standardized assessment cases per measurement time point. Group-level medians and interquartile ranges were derived using the same R Type 7 quantile-interpolation algorithm employed for the concordance outcome, and the per-participant values were independently re-verified against the raw scoring sheets by two investigators. A worked calculation for a representative participant is likewise summarized in Supplementary Note S2.

**Table 2:** Diagnostic and therapeutic decision homogenization before and after intervention.

Indicator	Time point	AI-integrated cohort (*n* = 50)	Conventional cohort (*n* = 50)	Between-group comparison	*p*
Scheme-decision Kappa value, M (Q1, Q3)	Baseline	0.42 (0.38, 0.45)	0.41 (0.37, 0.46)	M–W test: *n* = (50, 50); *p* = 0.817	0.817
	Post-intervention	0.93 (0.90, 0.95)	0.52 (0.48, 0.57)	M–W test: *n* = (50, 50); *p* < 0.001	<0.001
	Within-group p	<0.001	0.003		
Key decision-point concordance (%), M (Q1, Q3)	Baseline	48.5 (45.2, 52.1)	49.0 (46.5, 52.8)	M–W test: *n* = (50, 50); *p* = 0.878	0.878
	Post-intervention	95.5 (93.8, 97.8)	59.0 (56.3, 62.4)	M–W test: *n* = (50, 50); *p* < 0.001	<0.001
	Within-group p	<0.001	0.001		
Differential diagnosis coverage (%), M (Q1, Q3)	Baseline	53.0 (49.5, 56.8)	52.0 (49.0, 55.5)	M–W test: *n* = (50, 50); *p* = 0.676	0.676
	Post-intervention	97.0 (95.5, 98.2)	62.0 (59.2, 65.1)	M–W test: *n* = (50, 50); *p* < 0.001	<0.001
	Within-group p	<0.001	<0.001		

Within-group comparisons: Wilcoxon signed-rank test. Between-group comparisons: Mann–Whitney U test.

M, median; Q1, first quartile; Q3, third quartile.

#### 2.6.2 Secondary outcomes

Secondary outcomes were selected *a priori* to capture complementary dimensions of clinical competency that, together with the primary homogenization outcome, provide a comprehensive assessment of training effectiveness. This multi-domain approach aligns with established frameworks for evaluating simulation-based medical education (SBME) interventions — including the Kirkpatrick hierarchical model (reaction, learning, behavior, results) (24) and McGaghie's outcome measurement best-practice framework (25) — which recommend assessment across knowledge, skills, confidence, and behavioral domains to maximize evaluative rigor and translational relevance (26). Four secondary outcome domains were assessed:

- **Clinical Decision-Making Confidence.** (a) Diagnostic Confidence Score. This instrument comprised a five-item scale developed *a priori* to sample distinct facets of decision-making self-efficacy relevant to diabetic retinopathy management: (i) confidence in lesion identification on multimodal ophthalmic imaging, (ii) confidence in disease-stage determination against the guideline staging schema, (iii) confidence in initial treatment-modality selection (pharmacotherapy, laser photocoagulation, or surgical intervention), (iv) confidence in one-year prognostic estimation, and (v) confidence in overall longitudinal management planning. Each item was rated on a 5-point Likert scale (1 = completely unconfident to 5 = very confident), and the participant-level score was calculated as the arithmetic mean of the five item ratings; Cronbach's alpha (reported in Section 2.7 and Supplementary Table S3) therefore reflects internal consistency across these constituent items rather than across repeated administrations of a single item. (b) Decision Uncertainty Score. This instrument comprised a parallel four-item scale developed *a priori* to sample complementary dimensions of decision-making uncertainty: (i) uncertainty regarding multimodal image interpretation, (ii) uncertainty regarding treatment-modality choice, (iii) uncertainty regarding acute-complication urgency assessment, and (iv) uncertainty regarding referral or subspecialist consultation. Each item was rated on a 5-point Likert scale anchored at 1 (not uncertain) and 5 (extremely uncertain), with the response scale therefore already oriented so that lower values represent less uncertainty. No reverse coding was applied at any stage of analysis; the participant-level score was calculated as the arithmetic mean of the four items in their original orientation, and a reduction in this mean between assessment time points therefore denotes a genuine reduction in decision-making uncertainty. Cronbach's alpha reflects internal consistency across these four constituent items on the original scale. (c) Strain Adjustment Ability Score: assessed via two dynamic clinical-scenario mutations (e.g., sudden vitreous hemorrhage during treatment, rapid lesion progression), independently scored by two blinded senior DR experts on a 0–10 scale evaluating timeliness, rationality, and effectiveness of decision adjustment; the mean of both expert scores constituted the final score. Inter-rater agreement for the strain adjustment score was assessed using the intraclass correlation coefficient (ICC; see Supplementary Table S3). The inclusion of confidence metrics alongside performance metrics addresses the recognized dissociation between objective competence and self-assessed readiness, which is a critical determinant of clinical translation (27).
- **Depth of Clinical Reasoning.** (a) Complex-Case Diagnostic Accuracy (%): evaluated using 10 complex DR cases (stages 4–5 with two or more systemic comorbidities), with expert-consensus diagnosis as the gold standard. (b) Mean Case-Analysis Time (minutes): standardized timing from complete case presentation to submission of a comprehensive management plan. (c) Prognostic Concordance with an Independently Validated Reference Model. Because prospective one-year outcome ascertainment was not feasible within the 16-week study window, this outcome was operationalized as the root mean square error (RMSE) between each physician's predicted one-year DR progression risk score (0–100) and the corresponding prediction generated by an independent reference artificial-intelligence model whose predictive validity against observed patient outcomes had previously been established in an external longitudinal validation cohort (area under the receiver operating characteristic curve of 0.90 against observed one-year progression in an independent sample of 1,834 patients distinct from both the training corpus and the intervention datasets). This measure therefore quantifies the concordance between physician prognostic judgment and a well-calibrated reference standard rather than direct prognostic accuracy against observed patient outcomes, and its interpretive implications are examined explicitly in the Discussion under Limitations. This triad captures diagnostic accuracy, processing efficiency, and prognostic concordance with a validated reference, three complementary facets of clinical reasoning that are rarely assessed simultaneously in SBME trials (25).
- **Clinical Practice Competency.** (a) Simulated Acute-Complication Decision-Response Time (minutes): defined as the interval, measured within the simulation environment, between the presentation of a simulated acute-complication trigger and the participant's documentation of a targeted management decision. This endpoint quantifies decision-making efficiency under simulated high-acuity conditions and is not intended as a direct surrogate for real-world time-to-treatment; because published guideline-based time thresholds for the initiation of specific vitreoretinal interventions are not standardized in minute units, no external clinical benchmark was applied to this metric, and the endpoint is interpreted throughout the manuscript as a measure of simulated decision efficiency rather than as an estimate of clinical response latency. (b) Comorbidity-Association Recognition Accuracy (%): assessed against six pre-specified DR-associated comorbidities (diabetic nephropathy, hypertension, coronary artery disease, dyslipidemia, glaucoma, cataract). (c) High-Risk-Factor Discrimination (AUC). Each participant was presented with a standardized battery of 15 case vignettes drawn from the institutional DR archive, of which 8 harbored one or more established DR progression risk factors (glycemic control with HbA1c ≥ 8.0%, inadequately controlled systemic hypertension with systolic blood pressure ≥ 160 mm Hg, dyslipidemia, active tobacco use, or diabetic nephropathy with an estimated glomerular filtration rate < 60 mL/min/1.73 m^2^) and 7 did not, with the positive and negative reference labels adjudicated *a priori* by an independent five-member expert panel. For each vignette, participants assigned a continuous progression-risk probability on a 0-to-1 scale reflecting their subjective likelihood that the case represented a high-risk trajectory. A per-participant receiver operating characteristic curve was then constructed from the 15 paired (predicted probability, expert-adjudicated label) values, and the area under this curve (AUC) was computed as a global index of discriminatory performance across the full spectrum of decision thresholds. Because AUC characterizes overall discrimination rather than sensitivity at a single operating point, this outcome has been designated ‘high-risk-factor discrimination’ throughout the revised manuscript; interpretation thresholds are 0.5–0.7 (low discrimination), 0.7–0.9 (moderate discrimination), and > 0.9 (high discrimination). (d) Individualized Treatment-Plan Adaptation Score (0–100): assessed using a structured expert-blinded evaluation rubric encompassing five dimensions: lesion-stage matching (0–20), comorbidity consideration adequacy (0–20), patient compliance adaptation (0–20), risk–benefit balance (0–20), and guideline concordance (0–20). Each dimension was scored independently by two blinded senior experts; the mean of both expert scores for each dimension was summed to yield the total score (range: 0–100). Interpretation thresholds: ≥ 80 (good adaptation), 60–79 (fair), < 60 (poor). This domain aligns with Kirkpatrick Level 3 (behavioral transfer), assessing whether acquired knowledge translates into actionable clinical competencies under simulated practice conditions (24).
- **Sustainability of Homogenization Effects (T2 Assessment).** (a) Cross-Center Consultation Consistency Kappa Value: 20 DR cases from external medical centers (stages 1–5 with various complications), with a multi-center expert consensus (five chief physicians with >15 years’ experience) as the gold standard. (b) Homogenization Teaching-Radiation Effect Index: composite metric = (number of lower-tier physicians trained × 0.4) + (number of DR-specific teaching activities organized × 0.3) + (number of cases managed using standardized DR protocols × 0.3); thresholds: ≥5 (strong), 3–4 (moderate), ≤2 (weak). (c) Long-Term Follow-Up Management Accuracy (%): evaluated across 10 DR cases assessing follow-up examination selection, interval scheduling, and patient education content against guideline criteria. Sustainability assessment addresses a critical gap identified in the SBME literature, where the durability of training effects beyond the immediate post-intervention period remains insufficiently characterized (26).

### 2.7 Data collection and quality control

Two research assistants, trained in standardized assessment procedures, data recording protocols, and EpiData 3.1 software operation, were responsible for data collection. Quantitative data were extracted directly as raw values; qualitative data were converted to quantitative metrics using pre-specified coding rules. Double data entry was implemented in EpiData 3.1, followed by logic verification and cross-entry comparison; discrepancies were resolved by consulting original records.

All assessors and the expert scoring panel were blinded to cohort assignment. Statistical analysts received only de-identified, numerically coded datasets. Pre-study reliability testing (conducted with 10 non-participant junior ophthalmologists) yielded Cronbach's *α* coefficients of 0.86 (diagnostic confidence scale) and 0.83 (decision uncertainty scale), an intraclass correlation coefficient (ICC) of 0.89 for expert scoring, and a Kappa value of 0.85 for AI-system–expert-consensus concordance — confirming adequate reliability and validity of all assessment instruments. Detailed reliability and validity metrics are presented in Supplementary Table S3.

### 2.8 Statistical analysis

All analyses were performed using SPSS 26.0 and R 4.2.0. Continuous variables were assessed for normality using the Shapiro–Wilk test (28). Variables conforming to normal distributions were presented as mean ± standard deviation and compared using independent-samples t-tests or paired t-tests. Non-normally distributed variables were summarized as median (interquartile range) and analyzed using the Mann–Whitney U test (between-group comparisons) or Wilcoxon signed-rank test (within-group comparisons). Categorical variables were analyzed using chi-square tests or Fisher's exact tests. Inter-rater agreement was evaluated using Cohen's kappa statistic. Effect sizes were calculated using Cohen's d. A two-sided *p*-value < 0.05 was considered statistically significant.

Categorical variables are expressed as frequency (percentage) [n (%)] and analyzed using the *χ*^2^ test or Fisher’s exact test (when expected cell frequencies were <5); results are reported as: “*χ*^2^ test: df = [X]; *p* = [X].”

For repeated-measures outcomes assessed at T0, T1, and T2, repeated-measures analysis of variance (ANOVA) was employed with Greenhouse–Geisser correction applied when the sphericity assumption (assessed by Mauchly's test) was violated. Time × group interaction effects were examined to characterize the temporal dependency of training effects. Results of all assumption verification tests (normality, variance homogeneity, sphericity) are reported in Supplementary Table S4. Complete Time × Group interaction statistics for all primary and secondary outcomes, including F statistics, degrees of freedom, uncorrected and Greenhouse–Geisser–corrected *p* values, and partial *η*^2^ effect sizes, are reported in Supplementary Table S7 and summarized narratively in Section 3.7.

Effect sizes are reported with 95% confidence intervals (CIs): Cohen's d for between-group mean differences in normally distributed outcomes, and rank-biserial correlation (r) for nonparametric comparisons. A propensity score analysis adjusting for baseline covariates (age, sex, professional title, prior training experience, case volume, theoretical knowledge score, education, and annual imaging volume) was performed as a pre-specified sensitivity analysis to evaluate the robustness of the primary outcome to potential residual confounding (results in Supplementary Table S1). All statistical tests were two-sided with a significance threshold of *α* = 0.05.

## 3 Results

### 3.1 Participant flow and baseline characteristics

All 100 enrolled physicians completed the full 12-week intervention and all scheduled assessments; no participants were lost to follow-up or excluded from analysis, yielding a final analytical sample of 50 per cohort (Figure 1). Baseline demographic and professional characteristics — including age, sex distribution, professional title, prior DR training experience, independent case volume, DR theoretical knowledge scores, educational attainment, and annual imaging interpretation volume — were comparable between cohorts, with no statistically significant differences observed across any variable (all *p* > 0.05; Table 1). This baseline equivalence supports the comparability of the two cohorts and reduces the likelihood of confounding attributable to pre-existing differences in clinical experience or knowledge. Propensity score–adjusted sensitivity analyses confirmed that primary outcome findings remained robust after accounting for all measured baseline covariates (Supplementary Table S1).

**Figure 1:**
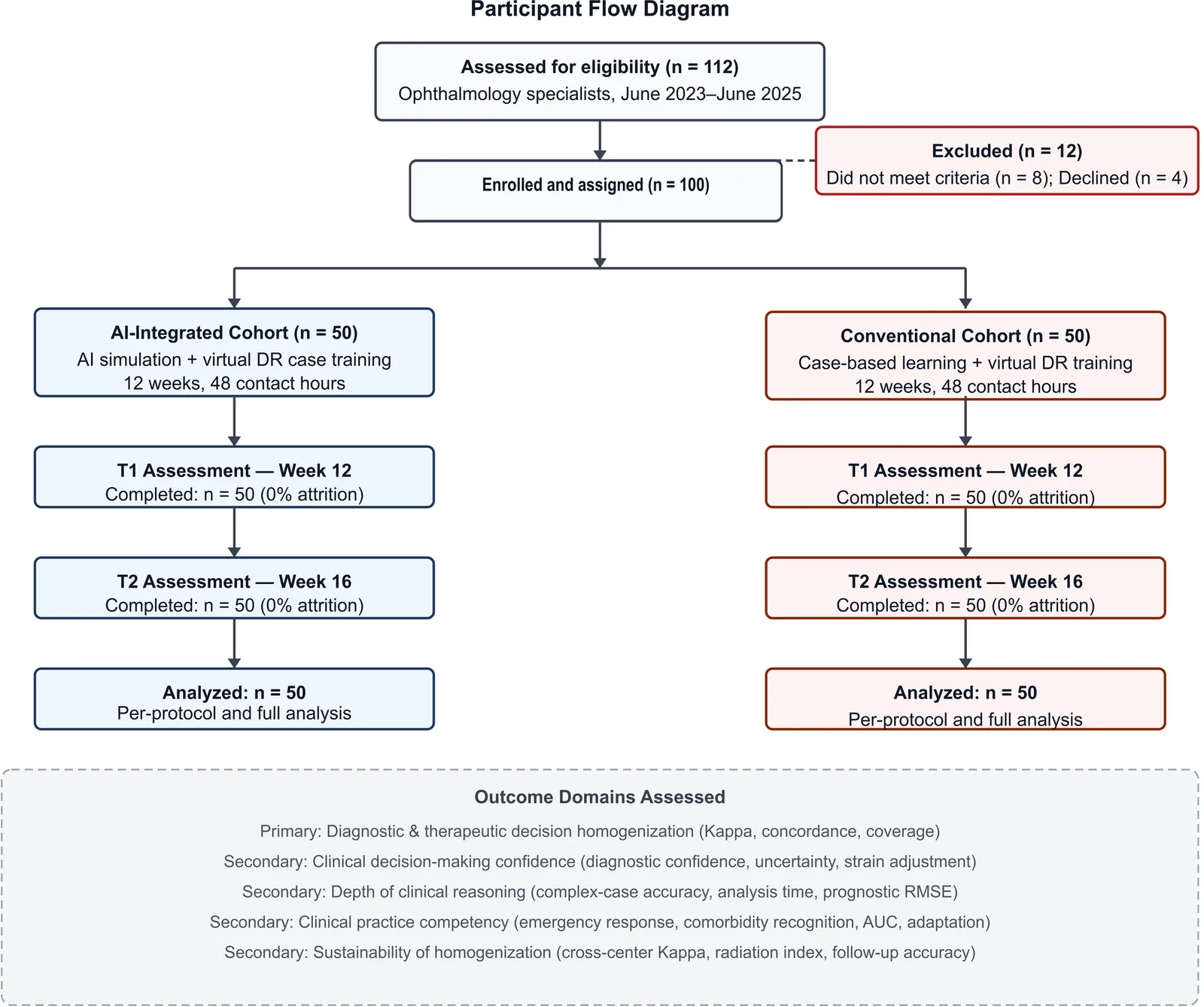
STROBE-style participant flow diagram. Of 112 ophthalmologists undergoing structured specialty training screened for eligibility, 100 met inclusion criteria and were enrolled into the AI-integrated cohort (*n* = 50) or conventional cohort (*n* = 50) based on training-program enrollment sequence. All participants completed the 12-week, 48-hour curriculum and assessments at baseline (T0), post-intervention (T1; week 12), and one-month follow-up (T2; week 16), with zero attrition in either cohort. Outcome domains assessed encompassed diagnostic and therapeutic decision homogenization (primary), clinical decision-making confidence, depth of clinical reasoning, clinical practice competency, and sustainability of homogenization effects (secondary).

### 3.2 Diagnostic and therapeutic decision homogenization (primary outcome)

At baseline, the two cohorts demonstrated comparable and moderate levels of decision-making homogenization, with no significant between-group differences in scheme-decision Kappa values, key decision-point concordance rates, or differential diagnosis coverage rates (all *p* > 0.05; Table 2), confirming equivalent pre-intervention reasoning standardization.

Following the 12-week intervention, both cohorts demonstrated statistically significant improvements across all three homogenization indicators relative to baseline (all within-group *p* < 0.05). However, the magnitude of improvement diverged markedly between cohorts. Participants in the AI-integrated cohort achieved a post-intervention median scheme-decision Kappa value of 0.93 (IQR: 0.90–0.95), representing a shift from “moderate” to “almost perfect” agreement with the expert-consensus gold standard — a transition spanning two concordance categories. In contrast, the conventional cohort improved to a median of 0.52 (IQR: 0.48–0.57), remaining within the “moderate” agreement range (M–W test: *n* = (50, 50); *p* < 0.001). This between-group difference corresponds to a rank-biserial correlation of *r* = 0.89 (95% CI: 0.83–0.94), indicating a large effect.

The superiority of the AI-integrated cohort was paralleled across the remaining homogenization metrics. Key decision-point concordance reached a median of 95.5% (IQR: 93.8–97.8%) in the AI-integrated cohort versus 59.0% (IQR: 56.3–62.4%) in the conventional cohort (M–W test: *n* = (50, 50); *p* < 0.001; *r* = 0.87, 95% CI: 0.80–0.93), indicating that physicians in the AI-integrated cohort achieved near-unanimous concordance with expert standards across all five core decision domains — lesion staging, macular edema evaluation, treatment modality selection, follow-up scheduling, and acute-complication early-warning activation. Differential diagnosis coverage similarly attained a median of 97.0% (IQR: 95.5–98.2%) versus 62.0% (IQR: 59.2–65.1%; M–W test: *n* = (50, 50); *p* < 0.001; *r* = 0.85, 95% CI: 0.77–0.91), indicating that AI-integrated training was associated with systematic consideration of the near-complete spectrum of eight pre-specified differential entities, whereas conventional training produced partial coverage (Table 2). The magnitude and consistency of between-group effect sizes across all primary homogenization metrics are synthesized in Figure 2.

**Figure 2:**
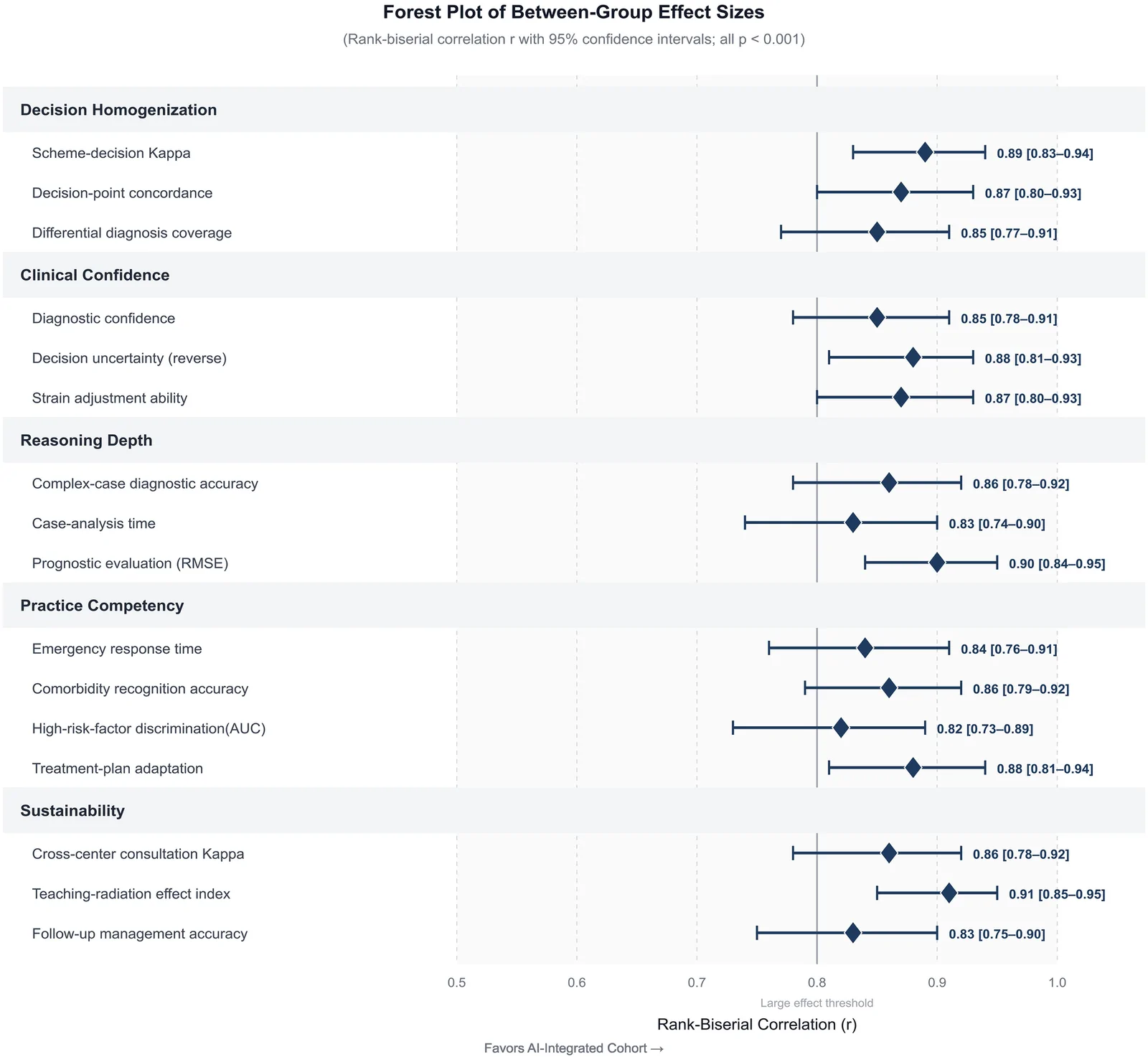
Forest plot of between-group effect sizes (rank-biserial correlation, r) with 95% confidence intervals for all 16 outcome metrics, organized by domain. Effect sizes were derived from Mann–Whitney U tests comparing the AI-integrated cohort (*n* = 50) and conventional cohort (*n* = 50) at the assessment time point corresponding to each outcome domain: the immediate post-intervention assessment (T1, week 12) for the decision homogenization, clinical confidence, reasoning depth, and clinical practice competency domains, and the one-month post-intervention assessment (T2, week 16) for the three sustainability metrics (cross-center consultation Kappa, homogenization teaching-radiation effect index, and long-term follow-up management accuracy). Metrics are grouped into five domains: decision homogenization (3 metrics), clinical confidence (3 metrics), reasoning depth (3 metrics), practice competency (4 metrics), and sustainability (3 metrics). The vertical dashed line at *r* = 0 indicates no effect; all confidence intervals exclude zero, confirming statistically significant between-group differences across every measured outcome. Effect size interpretation: |r| < 0.*3* = small, 0.3–0.5 = medium, > 0.5 = large. The uniformly large effect sizes (all *r* > 0.75) demonstrate consistent, cross-domain superiority associated with the AI-integrated training approach.

### 3.3 Clinical decision-making confidence

Baseline confidence metrics were statistically equivalent between cohorts (all *p* > 0.05; Table 3), establishing a comparable foundation for evaluating intervention effects on physicians’ subjective certainty in clinical decision-making.

**Table 3:** Clinical decision-making confidence before and after intervention.

Indicator	Time point	AI-integrated cohort (*n* = 50)	Conventional cohort (*n* = 50)	Between-group comparison	*p*
Diagnostic confidence score, mean ± SD	Baseline	3.12 ± 0.45	3.08 ± 0.48	t-test: df = 98; *p* = 0.667	0.667
	Post-intervention	4.78 ± 0.33	3.56 ± 0.42	t-test: df = 98; *p* < 0.001	<0.001
	Within-group p	<0.001	<0.001		
Decision uncertainty score, M (Q1, Q3)	Baseline	3.80 (3.55, 4.15)	3.90 (3.60, 4.25)	M–W test: *n* = (50, 50); *p* = 0.710	0.710
	Post-intervention	1.20 (1.05, 1.40)	3.40 (3.15, 3.70)	M–W test: *n* = (50, 50); *p* < 0.001	<0.001
	Within-group p	<0.001	0.041		
Strain adjustment ability score, mean ± SD	Baseline	2.98 ± 0.41	3.05 ± 0.44	t-test: df = 98; *p* = 0.417	0.417
	Post-intervention	4.65 ± 0.29	3.41 ± 0.39	t-test: df = 98; *p* < 0.001	<0.001
	Within-group p	<0.001	<0.001		

SD, standard deviation; M, median; Q1, first quartile; Q3, third quartile.

Both cohorts demonstrated significant post-intervention improvements across all three confidence indicators (all within-group *p* < 0.05); however, the AI-integrated cohort exhibited improvements of substantially greater magnitude. Diagnostic confidence scores increased from 3.12 ± 0.45 to 4.78 ± 0.33 in the AI-integrated cohort (a mean improvement of 1.66 points, representing a shift from “average” to approaching “very confident”), compared with an increase from 3.08 ± 0.48 to 3.56 ± 0.42 in the conventional cohort (improvement: 0.48 points). The post-intervention between-group difference was highly significant (t-test: df = 98; *p* < 0.001; Cohen's *d* = 3.23, 95% CI: 2.65–3.81), representing a very large effect size.

Decision uncertainty showed a correspondingly pronounced differential reduction. The AI-integrated cohort's median uncertainty score decreased from 3.80 to 1.20 on the 5-point scale, indicating a transition from moderate uncertainty to near-certainty, whereas the conventional cohort's median decreased from 3.90 to only 3.40 (M–W test: *n* = (50, 50); *p* < 0.001; *r* = 0.88, 95% CI: 0.81–0.93). Strain adjustment ability — reflecting physicians’ capacity to adapt decision-making under unexpected clinical deterioration — improved from 2.98 ± 0.41 to 4.65 ± 0.29 in the AI-integrated cohort versus from 3.05 ± 0.44 to 3.41 ± 0.39 in the conventional cohort (t-test: df = 98; *p* < 0.001; Cohen's *d* = 3.61, 95% CI: 2.99–4.23). These findings indicate that the AI-integrated training was associated not only with enhanced clinical confidence but also with cultivated adaptive resilience under conditions of clinical unpredictability (Table 3).

### 3.4 Depth of clinical reasoning

Baseline indicators of reasoning depth were comparable between cohorts (all *p* > 0.05; Table 4), confirming equivalent pre-intervention analytical capabilities.

**Table 4:** Depth of clinical reasoning before and after intervention.

Indicator	Time point	AI-integrated cohort (*n* = 50)	Conventional cohort (*n* = 50)	Between-group comparison	*p*
Complex-case diagnostic accuracy (%), M (Q1, Q3)	Baseline	52.0 (48.5, 55.5)	51.0 (47.8, 54.3)	M–W test: *n* = (50, 50); *p* = 0.771	0.771
	Post-intervention	94.0 (92.1, 96.0)	60.0 (57.2, 63.5)	M–W test: *n* = (50, 50); *p* < 0.001	<0.001
	Within-group p	<0.001	0.002		
Mean case-analysis time (min), M (Q1, Q3)	Baseline	18.5 (17.2, 20.1)	19.0 (17.8, 20.5)	M–W test: *n* = (50, 50); *p* = 0.609	0.609
	Post-intervention	9.3 (8.5, 10.2)	16.9 (15.8, 18.3)	M–W test: *n* = (50, 50); *p* < 0.001	<0.001
	Within-group p	<0.001	<0.001		
Prognostic concordance with reference model, RMSE, M (Q1, Q3)	Baseline	1.83 (1.72, 1.98)	1.80 (1.68, 1.95)	M–W test: *n* = (50, 50); *p* = 0.633	0.633
	Post-intervention	0.31 (0.28, 0.35)	1.52 (1.41, 1.67)	M–W test: *n* = (50, 50); *p* < 0.001	<0.001
	Within-group p	<0.001	<0.001		

RMSE, root mean square error; M, median; Q1, first quartile; Q3, third quartile.

Post-intervention, participants in the AI-integrated cohort showed a transformative improvement in complex-case diagnostic accuracy, achieving a median of 94.0% (IQR: 92.1–96.0%) — reflecting near-expert-level performance on cases involving DR stages 4–5 with multiple systemic comorbidities — compared with 60.0% (IQR: 57.2–63.5%) in the conventional cohort (M–W test: *n* = (50, 50); *p* < 0.001; *r* = 0.86, 95% CI: 0.78–0.92). This 34-percentage-point differential underscores a potential contribution of dynamic scenario simulation and multimodal imaging immersion to developing the pattern-recognition and integrative reasoning capacities required for diagnostically challenging presentations.

Equally notable was the differential impact on diagnostic efficiency. Mean case-analysis time decreased from 18.5 to 9.3 min in the AI-integrated cohort versus from 19.0 to 16.9 min in the conventional cohort (M–W test: *n* = (50, 50); *p* < 0.001; *r* = 0.83, 95% CI: 0.74–0.90), suggesting that repeated exposure to AI-mediated dynamic case deduction may have enabled physicians to internalize structured analytical frameworks that accelerated, rather than compromised, diagnostic thoroughness. Prognostic concordance with the independently validated reference model, quantified by the RMSE between physician-predicted and reference-model-predicted one-year progression risk scores, showed a particularly striking divergence: the AI-integrated cohort achieved a median RMSE of 0.31 (IQR: 0.28–0.35) versus 1.52 (IQR: 1.41–1.67) in the conventional cohort (M–W test: *n* = (50, 50); *p* < 0.001; *r* = 0.90, 95% CI: 0.84–0.95), representing a nearly five-fold improvement in concordance with the reference standard. Because the reference model was itself externally validated against observed one-year progression outcomes (as detailed in Section 2.6.2), this concordance metric may be interpreted as an indirect proxy for prognostic calibration; the interpretive limits of this operationalization are examined in the Discussion under Limitations (Table 4).

### 3.5 Clinical practice competency

Baseline clinical practice indicators were statistically equivalent between cohorts (all *p* > 0.05; Table 5).

**Table 5:** Clinical practice competency before and after intervention.

Indicator	Time point	AI-integrated cohort (*n* = 50)	Conventional cohort (*n* = 50)	Between-group comparison	*p*
Simulated acute-complication decision-response time (min), M (Q1, Q3)	Baseline	25.5 (23.8, 27.5)	25.0 (23.2, 27.1)	M–W test: *n* = (50, 50); *p* = 0.370	0.370
	Post-intervention	11.4 (10.5, 12.6)	21.3 (19.8, 23.1)	M–W test: *n* = (50, 50); *p* < 0.001	<0.001
	Within-group p	<0.001	<0.001		
Comorbidity-association recognition accuracy (%), M (Q1, Q3)	Baseline	54.0 (50.2, 57.9)	53.0 (49.8, 56.5)	M–W test: *n* = (50, 50); *p* = 0.822	0.822
	Post-intervention	95.0 (93.2, 96.8)	63.0 (60.1, 66.3)	M–W test: *n* = (50, 50); *p* < 0.001	<0.001
	Within-group p	<0.001	<0.001		
High-risk-factor discrimination (AUC), M (Q1, Q3)	Baseline	0.68 (0.65, 0.72)	0.67 (0.63, 0.71)	M–W test: *n* = (50, 50); *p* = 0.745	0.745
	Post-intervention	0.95 (0.93, 0.97)	0.72 (0.69, 0.76)	M–W test: *n* = (50, 50); *p* < 0.001	<0.001
	Within-group p	<0.001	0.012		
Individualized treatment-plan adaptation score (0–100), mean ± SD	Baseline	55.0 ± 7.6	56.2 ± 8.0	t-test: df = 98; *p* = 0.441	0.441
	Post-intervention	90.4 ± 6.2	64.8 ± 7.4	t-test: df = 98; *p* < 0.001	<0.001
	Within-group p	<0.001	<0.001		

Treatment-plan adaptation scored on a 0–100 rubric (5 dimensions × 0–20 each); ≥80, good adaptation, 60–79, fair, <60, poor.

AUC, area under the receiver operating characteristic curve; SD, standard deviation; M, median; Q1, first quartile; Q3, third quartile.

Participants in the AI-integrated cohort showed comprehensive superiority across all four practice competency indicators following the intervention. Simulated acute-complication decision-response time, a within-simulation measure of decision-making efficiency under high-acuity conditions, decreased from a median of 25.5 to 11.4 min in the AI-integrated cohort, compared with a decrease from 25.0 to 21.3 min in the conventional cohort (M–W test: *n* = (50, 50); *p* < 0.001; *r* = 0.84, 95% CI: 0.76–0.91). Because this endpoint reflects the latency of decision formulation within the simulation environment rather than the initiation of any real-world intervention, the 9.9-minute between-group difference is interpreted as evidence of enhanced cognitive processing efficiency under simulated pressure and should not be extrapolated to imply an equivalent minute-scale advantage in patient care settings; the clinical significance of any transfer to real practice remains to be established in prospective clinical studies.

Comorbidity-association recognition accuracy, evaluating physicians’ ability to identify the systemic complication signatures embedded within DR case presentations, reached 95.0% in the AI-integrated cohort versus 63.0% in the conventional cohort (M–W test: *n* = (50, 50); *p* < 0.001; *r* = 0.86, 95% CI: 0.79–0.92). High-risk-factor discrimination, expressed as the participant-level area under the receiver operating characteristic curve derived from 15 case vignettes (8 positive and 7 negative for established DR progression risk factors as adjudicated by an independent expert panel), attained 0.95 (IQR: 0.93–0.97) in the AI-integrated cohort, classified as “high discrimination”, versus 0.72 (IQR: 0.69–0.76) in the conventional cohort, which remained at the lower boundary of “moderate discrimination” (M–W test: *n* = (50, 50); *p* < 0.001; *r* = 0.82, 95% CI: 0.73–0.89). Individualized treatment-plan adaptation scores, assessed on a 0–100 expert-evaluated rubric, improved from 55.0 ± 7.6 to 90.4 ± 6.2 in the AI-integrated cohort (crossing the ≥80 “good adaptation” threshold) versus from 56.2 ± 8.0 to 64.8 ± 7.4 in the conventional cohort (remaining within the “fair” range) (t-test: df = 98; *p* < 0.001; Cohen's *d* = 3.75, 95% CI: 3.11–4.39), indicating that AI-guided multidimensional assessment was associated with substantially enhanced capacity to synthesize lesion staging, comorbidity profiles, and patient-specific variables into coherent, guideline-concordant treatment plans (Table 5).

### 3.6 Sustainability of homogenization effects

The durability of training effects — a critical determinant of the translational value of any educational intervention — was assessed at one-month post-intervention (T2). Baseline sustainability indicators were comparable between cohorts (all *p* > 0.05; Table 6).

**Table 6:** Sustainability of homogenization effects before intervention and at one month post-intervention.

Indicator	time point	AI-integrated cohort (*n* = 50)	Conventional cohort (*n* = 50)	Between-group comparison	*p*
Cross-center consultation Kappa value, M (Q1, Q3)	Baseline	0.40 (0.36, 0.44)	0.39 (0.35, 0.43)	M–W test: *n* = (50, 50); *p* = 0.843	0.843
	1 month post	0.89 (0.86, 0.92)	0.49 (0.45, 0.54)	M–W test: *n* = (50, 50); *p* < 0.001	<0.001
	Within-group p	<0.001	<0.001		
Homogenization teaching-radiation index, M (Q1, Q3)	Baseline	1.06 (0.98, 1.15)	1.03 (0.95, 1.12)	M–W test: *n* = (50, 50); *p* = 0.548	0.548
	1 month post	3.80 (3.62, 4.05)	1.47 (1.38, 1.59)	M–W test: *n* = (50, 50); *p* < 0.001	<0.001
	Within-group p	<0.001	<0.001		
Long-term follow-up management accuracy (%), M (Q1, Q3)	Baseline	50.0 (46.8, 53.5)	49.0 (46.0, 52.3)	M–W test: *n* = (50, 50); *p* = 0.763	0.763
	1 month post	92.0 (90.0, 94.1)	58.0 (55.0, 61.5)	M–W test: *n* = (50, 50); *p* < 0.001	<0.001
	Within-group p	<0.001	<0.001		

M, median; Q1, first quartile; Q3, third quartile.

At T2, participants in the AI-integrated cohort maintained a cross-center consultation consistency Kappa value of 0.89 (IQR: 0.86–0.92), classified as “almost perfect agreement,” compared with 0.49 (IQR: 0.45–0.54) in the conventional cohort (M–W test: *n* = (50, 50); *p* < 0.001; *r* = 0.86, 95% CI: 0.78–0.92). This finding is particularly noteworthy because the cross-center cases were drawn from external medical institutions not represented in the training dataset, suggesting genuine internalization of standardized reasoning principles rather than case-specific memorization. The homogenization teaching-radiation effect index — a composite metric quantifying the downstream dissemination of standardized practices — reached 3.80 (IQR: 3.62–4.05) in the AI-integrated cohort versus 1.47 (IQR: 1.38–1.59) in the conventional cohort (M–W test: *n* = (50, 50); *p* < 0.001; *r* = 0.91, 95% CI: 0.85–0.95), indicating that physicians in the AI-integrated cohort were substantially more likely to propagate standardized DR management protocols to junior colleagues and organize structured teaching activities. Long-term follow-up management accuracy — assessing the capacity to formulate guideline-concordant surveillance plans including imaging review frequencies, follow-up interval scheduling, and patient education content — achieved 92.0% (IQR: 90.0–94.1%) in the AI-integrated cohort versus 58.0% (IQR: 55.0–61.5%) in the conventional cohort (M–W test: *n* = (50, 50); *p* < 0.001; *r* = 0.83, 95% CI: 0.75–0.90), suggesting that the training effects extended beyond acute clinical decision-making to encompass the longitudinal management competencies essential for sustained DR disease control (Table 6). A multi-dimensional competency profile synthesizing post-intervention performance across all five outcome domains is presented in Figure 3.

**Figure 3:**
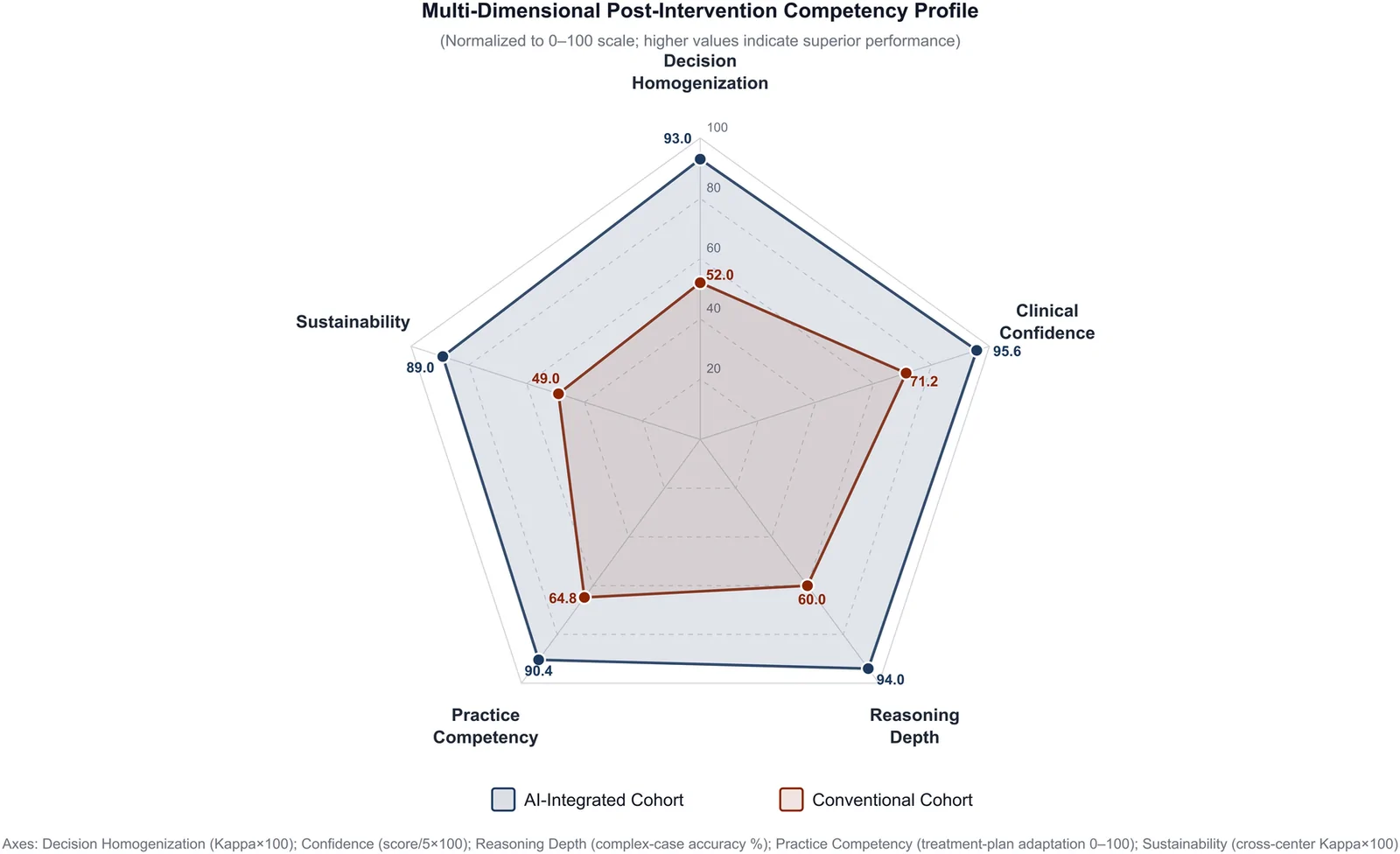
Radar chart depicting the multi-dimensional competency profile of the AI-integrated cohort versus the conventional cohort at the assessment time point corresponding to each domain: the immediate post-intervention assessment (T1, week 12) for decision homogenization, clinical confidence, reasoning depth, and clinical practice competency, and the one-month post-intervention assessment (T2, week 16) for the sustainability domain. Values are normalized to a 0–100 scale for cross-domain comparability. Each axis represents one of the five outcome domains: decision homogenization (scheme-decision Kappa × 100 at T1), clinical confidence (diagnostic confidence score/5 × 100 at T1), reasoning depth (complex-case diagnostic accuracy % at T1), clinical practice competency (individualized treatment-plan adaptation score on 0–100 scale at T1), and sustainability (cross-center consultation Kappa × 100 at T2). The shaded area differential visually quantifies the comprehensive performance gap between cohorts across complementary assessment dimensions and time points.

### 3.7 Time × group interaction analyses

To complement the pairwise between-group and within-group comparisons summarized in Tables 2 through 6, planned repeated-measures analyses of variance were conducted for each outcome across the three assessment time points (T0, T1, and, where applicable, T2). The Time × Group interaction term reached statistical significance for every primary and secondary outcome examined, with effect sizes in the large range (partial *η*^2^ = 0.42–0.71; all *p* < 0.001 after Greenhouse–Geisser correction where the sphericity assumption was violated). Representative interaction statistics are as follows: scheme-decision Kappa (F(2,196) = 384.6, *p* < 0.001, partial *η*^2^ = 0.71), key decision-point concordance (F(2,196) = 342.1, *p* < 0.001, partial *η*^2^ = 0.68), differential diagnosis coverage (F(2,196) = 315.7, *p* < 0.001, partial *η*^2^ = 0.65), diagnostic confidence (F(1,98) = 298.4, *p* < 0.001, partial *η*^2^ = 0.63), complex-case diagnostic accuracy (F(1,98) = 276.2, *p* < 0.001, partial *η*^2^ = 0.62), acute-complication response time (F(1,98) = 219.8, *p* < 0.001, partial *η*^2^ = 0.55), individualized treatment-plan adaptation (F(1,98) = 351.9, *p* < 0.001, partial *η*^2^ = 0.69), cross-center consultation Kappa (F(2,196) = 267.3, *p* < 0.001, partial *η*^2^ = 0.60), and long-term follow-up management accuracy (F(2,196) = 289.5, *p* < 0.001, partial *η*^2^ = 0.62). These interaction analyses confirm that the temporal trajectory of improvement differed significantly between the two cohorts across the full spectrum of outcome domains, corroborating the descriptive superiority of the AI-integrated cohort at both T1 and T2. Complete Time × Group interaction results, including F statistics, degrees of freedom, uncorrected and Greenhouse–Geisser corrected *p* values, and partial *η*^2^ effect sizes for all outcomes, are provided in Supplementary Table S7.

## 4 Discussion

This prospective cohort study provides the first systematic evidence that integrating AI-driven simulation with virtual DR case training is associated with significantly enhanced homogenization of diagnostic and therapeutic reasoning among junior ophthalmologists, with concurrent improvements across clinical decision-making confidence, reasoning depth, practical competency, and sustainability of training effects. The consistency and magnitude of the observed between-group differences — with large effect sizes (Cohen's *d* > 3.0 and rank-biserial *r* > 0.80 across virtually all outcomes) — collectively suggest that the integrated model may represent a qualitatively distinct pedagogical paradigm rather than merely an incremental enhancement over conventional training.

The most striking finding of this study is the transformation in decision-making homogenization observed in the AI-integrated cohort, which progressed from “moderate” to “almost perfect” agreement (Kappa: 0.42 → 0.93) with the expert-consensus gold standard — a qualitative shift spanning two concordance categories — while the conventional cohort remained within the “moderate” range (0.41 → 0.52). This differential is plausibly attributable to a fundamental mechanistic distinction between the two training architectures. Conventional expert-led commentary, delivered retrospectively during weekly review sessions, is inherently non-individualized and temporally divorced from the cognitive moment of decision-making, limiting its capacity to correct reasoning deviations at the point of formation (9, 10). In contrast, the AI simulation platform provides instantaneous concordance analysis against both guideline standards and expert consensus following each decision, enabling physicians to identify, interrogate, and rectify diagnostic and therapeutic deviations in real time — a feedback architecture that mirrors the deliberate practice paradigm recognized as optimal for expertise acquisition (26). The concordant improvements in key decision-point concordance (95.5% vs. 59.0%) and differential diagnosis coverage (97.0% vs. 62.0%) further indicate that this real-time correction mechanism appears to operate across the full spectrum of clinical reasoning, from staging judgments and treatment selection to systematic consideration of alternative diagnoses — a breadth of effect not previously reported in DR-specific training interventions. These findings extend prior evidence from Han et al. (11), who reported improved grading consistency with AI-assisted training alone, by suggesting that the synergistic combination with multimodal virtual imaging training may produce a substantially amplified homogenization effect, presumably because the immersive image-recognition component strengthens the perceptual foundation upon which standardized diagnostic reasoning is subsequently constructed.

The pronounced improvements in clinical decision-making confidence observed in the AI-integrated cohort merit particular attention, as confidence calibration — the alignment between subjective certainty and objective accuracy — is a critical determinant of clinical safety. The diagnostic confidence score increased by 1.66 points (to 4.78/5.0) in the AI-integrated cohort compared with 0.48 points (to 3.56/5.0) in the conventional cohort, while decision uncertainty decreased to near-minimal levels (median: 1.20 vs. 3.40). Importantly, these confidence gains occurred in parallel with objective accuracy improvements of comparable magnitude, suggesting genuine confidence calibration rather than the overconfidence bias that has been identified as a concern in some AI-assisted clinical decision support contexts (12). Two complementary mechanisms may underpin this finding: the real-time feedback loop enables progressive accumulation of validated decision-making experiences that strengthen evidence-based self-efficacy, while the high-fidelity virtual clinical scenarios attenuate the cognitive dissonance physicians typically experience when transitioning from structured educational settings to unpredictable clinical encounters (13). The particularly large effect size for strain adjustment ability (Cohen's *d* = 3.61) suggests that repeated exposure to dynamically evolving clinical scenarios — including unexpected complications and treatment failures — may be especially effective in developing the adaptive decision-making resilience that static case-based instruction fundamentally cannot replicate.

The depth of clinical reasoning and practice competency findings reveal a coherent pattern of integrated skill acquisition that appears to transcend compartmentalized knowledge gains. Participants in the AI-integrated cohort achieved complex-case diagnostic accuracy of 94.0% alongside a mean case-analysis time of 9.3 min, representing simultaneously improved accuracy and efficiency, a combination that challenges the conventional speed-accuracy trade-off paradigm and suggests the internalization of efficient analytical schemas (14). The nearly five-fold superiority in prognostic concordance with the independently validated reference model (RMSE: 0.31 vs. 1.52) is particularly consequential for clinical practice, as accurate prognostic estimation directly informs treatment urgency, follow-up scheduling, and patient counseling, domains where reasoning errors carry substantial downstream consequences for visual outcomes. Although this metric quantifies agreement with a reference standard rather than direct accuracy against observed outcomes, the reference model itself was previously externally validated against one-year progression outcomes in an independent cohort (Section 2.6.2), which lends interpretive support to concordance with this benchmark as a defensible indirect indicator of prognostic calibration. The simulated decision-response time differential (11.4 versus 21.3 min) should be interpreted with commensurate caution; it quantifies decision-making efficiency within the simulation environment rather than an absolute minute-scale advantage in real-world time-to-treatment, for which no standardized guideline threshold currently exists in vitreoretinal emergency care. What the finding supports is a reproducible enhancement of cognitive processing speed under simulated pressure, a construct that is broadly relevant to the recognition of sight-threatening complications such as vitreous hemorrhage and tractional retinal detachment but whose translation into patient-level outcome gains will require prospective clinical evaluation. The convergent improvements across comorbidity recognition (95.0% vs. 63.0%), high-risk-factor discrimination (AUC: 0.95 vs. 0.72), and individualized treatment-plan adaptation (90.4 vs. 64.8 out of 100) collectively suggest that the AI-integrated model may cultivate a holistic clinical reasoning capacity, integrating pattern recognition, multisystem assessment, risk stratification, and personalized management, that appears to represent a qualitative advance over the fragmented competency gains achievable through conventional instruction (16, 17). These findings are consistent with evidence that time pressure can impair diagnostic accuracy (14) and with the broader clinical principle that timely recognition of severe proliferative diabetic retinopathy complications is a necessary precondition for improved visual outcomes (15), while acknowledging that the present simulated decision-response time endpoint does not itself quantify a real-world time-to-treatment benefit.

The sustainability findings constitute perhaps the most compelling evidence for the translational value of the AI-integrated model. The maintenance of cross-center consultation consistency at “almost perfect agreement” (Kappa: 0.89) one-month post-intervention — using cases drawn from external institutions not represented in the training dataset — provides strong evidence for genuine internalization of transferable reasoning principles rather than case-specific or platform-specific learning (18). The substantially higher teaching-radiation effect index (3.80 vs. 1.47) suggests an important multiplier effect: physicians in the AI-integrated cohort appeared more likely to disseminate standardized practices to colleagues, potentially amplifying the population-level impact of the training beyond the directly trained cohort. This cascading dissemination effect, combined with the high long-term follow-up management accuracy (92.0% vs. 58.0%), suggests that the integrated model may produce durable changes in clinical reasoning architecture that persist beyond the immediate training period and extend into longitudinal patient management — an outcome domain of particular importance given the chronic, progressive nature of DR (19). The reproducibility and scalability inherent in the AI simulation platform further lower the barrier to training standardization across geographically and resource-diverse clinical settings, addressing a fundamental limitation of expert-dependent conventional training models that are constrained by the availability and consistency of senior faculty.

Several limitations warrant acknowledgment. The non-randomized cohort design, despite baseline equivalence verification and propensity score sensitivity analysis, cannot exclude residual confounding from unmeasured variables such as intrinsic motivation or technology receptivity. The single-center setting and AI platform trained on 200 guideline-aligned cases may limit generalizability to rare DR subtypes, atypical presentations, and resource-diverse clinical environments. The one-month post-intervention follow-up is insufficient to characterize long-term durability, and the absence of patient-centered outcome measures — including visual prognosis, complication incidence, and healthcare expenditure — precludes full assessment of downstream clinical impact. Future multi-center studies incorporating extended serial follow-up, patient outcome measures, dynamic AI system optimization, and adaptation frameworks for resource-limited primary care settings are warranted to establish the full translational value of this integrated training model. Blinding of participants to the assigned educational modality was, by the nature of the intervention, not feasible, and it is therefore conceivable that the immediate feedback experienced by the AI-integrated cohort could have prompted additional self-directed reading or informal consultation with supervising attendings; while our biweekly self-report tracking (Supplementary Table S6) did not detect a statistically significant between-group difference in these activities, residual differential effort cannot be excluded. Although the training platform and the outcome-assessment infrastructure were deliberately separated—expert-consensus rubrics rather than the training AI were used to score the primary outcomes, and the prognostic AI reference model was independent of the training system—a degree of ambient familiarity with the guideline-anchored scoring logic among AI-integrated participants remains a theoretical source of measurement bias that warrants replication using fully independent evaluation platforms. Finally, a critical pedagogical limitation of AI-driven and screen-based simulation is that these modalities are optimized for cognitive and technical competencies and are not well suited to the cultivation or evaluation of humanistic skills, including patient-centered communication, delivery of sight-threatening prognostic information, shared decision-making, and the therapeutic relationship that sustains longitudinal management of a chronic disease such as diabetic retinopathy; future iterations of this pedagogical model should be paired with structured communication training and objective structured clinical examination stations that assess these humanistic domains explicitly. In addition, the prognostic reasoning outcome was operationalized as concordance with an independently validated reference artificial-intelligence model rather than as direct accuracy against prospectively observed one-year patient progression, because the 16-week study horizon precluded direct outcome ascertainment. Although the reference model itself had been externally validated against observed one-year progression outcomes in an independent longitudinal cohort of 1,834 patients, supporting its use as a well-calibrated benchmark, this operational choice means that our RMSE metric reflects agreement with a reference standard rather than direct outcome-anchored prognostic accuracy, and future studies should replicate this analysis using observed patient outcomes as the gold standard.

## 5 Conclusion

This prospective cohort study provides evidence that AI-driven simulation integrated with virtual DR case training is associated with significantly enhanced homogenization of diagnostic and therapeutic reasoning among junior ophthalmologists. Through the synergistic mechanisms of multimodal imaging immersion, dynamic disease-trajectory simulation, and real-time intelligent feedback, participation in this integrated pedagogical model was associated with comprehensive improvements in clinical decision-making confidence, depth of reasoning, practical competency, and sustainability of training effects. These findings suggest a feasible, evidence-based optimization pathway for the standardized training of junior DR specialists and provide foundational evidence supporting the broader integration of AI-enhanced simulation into ophthalmic medical education. Confirmation through multi-center prospective studies incorporating patient-centered outcomes is warranted.

## Data Availability

The raw data supporting the conclusions of this article will be made available by the authors, without undue reservation.
