# Supplementary material for: Effect of AI-driven simulation integrated with virtual case-based training on diagnostic and therapeutic reasoning homogenization among junior ophthalmologists: a prospective cohort study
Source: Front Med (Lausanne). 2026 Sep 18;13:1812146. doi: 10.3389/fmed.2026.1812146 (PMC13630636; doi:10.3389/fmed.2026.1812146)
Supplement: Supplementary file 1 [file Datasheet1.docx]

**Supplementary Materials**

**Supplementary Table S1.** Propensity Score–Adjusted Sensitivity Analysis for Primary Outcomes

A propensity score was estimated using multivariable logistic regression incorporating all measured baseline covariates (age, sex, professional title, prior DR training experience, independent case volume, DR theoretical knowledge score, educational attainment, and annual imaging interpretation volume). The propensity score model demonstrated adequate balance (all standardized mean differences <0.10 after adjustment). Primary outcomes were re-analyzed using inverse probability of treatment weighting (IPTW).

| **Outcome** | **Unadjusted p** | **IPTW-Adjusted p** | **Conclusion** |
| --- | --- | --- | --- |
| Scheme-decision Kappa value (post-intervention) | <0.001 | <0.001 | Result robust |
| Key decision-point concordance (post-intervention) | <0.001 | <0.001 | Result robust |
| Differential diagnosis coverage (post-intervention) | <0.001 | <0.001 | Result robust |

All primary outcome findings remained statistically significant after propensity score adjustment, supporting the robustness of observed between-group differences to measured confounders.

**Supplementary Table S2.** Comparison of Training Components Across Cohorts

| **Component** | **AI-Integrated Cohort** | **Conventional Cohort** |
| --- | --- | --- |
| Total duration | 12 weeks, 48 hours | 12 weeks, 48 hours |
| Phase I content | DR guidelines (4 h) + AI-adaptive image recognition training (28 h) | DR guidelines (4 h) + Didactic image interpretation lectures (28 h) |
| Phase I feedback | Real-time AI + expert-consensus dual verification | Post-session expert commentary during lectures |
| Phase II content | Dynamic case simulation with branching disease evolution (8 h) | Static case-based group discussion + expert review (16 h) |
| Phase II feedback | Immediate decision-pathway analysis with guideline-concordance report | Weekly expert centralized commentary |
| Phase III content | Complex interdisciplinary case training with emergency scenarios (8 h) | Continued case discussion (included in Phase II hours) |
| Phase III feedback | Multidimensional AI-generated evaluation report | General improvement suggestions |
| Case materials | 200 de-identified cases (30% complex); dynamic evolution | Same cases; static presentation |
| Imaging modalities | Color fundus, FFA, OCT, OCTA (interactive) | Color fundus, FFA, OCT, OCTA (static review) |
| Virtual DR training | Identical across cohorts | Identical across cohorts |
| Teaching faculty | Same senior physicians (expert-consensus contributors) | Same senior physicians (expert-consensus contributors) |

DR = diabetic retinopathy; FFA = fundus fluorescein angiography; OCT = optical coherence tomography; OCTA = OCT angiography.

**Supplementary Table S3.** Reliability and Validity of Assessment Instruments

| **Instrument / Metric** | **Reliability Measure** | **Value** | **Interpretation** |
| --- | --- | --- | --- |
| Diagnostic confidence scale | Cronbach's α | 0.86 | Good internal consistency |
| Decision uncertainty scale | Cronbach's α | 0.83 | Good internal consistency |
| Expert scoring (strain adjustment ability) | ICC (two-way random, absolute agreement) | 0.89 | Excellent inter-rater reliability |
| Expert scoring (treatment-plan adaptation) | ICC (two-way random, absolute agreement) | 0.87 | Good inter-rater reliability |
| AI system vs. expert consensus | Kappa coefficient | 0.85 | Almost perfect agreement |
| Complex-case diagnostic gold standard | Inter-expert Kappa | 0.91 | Almost perfect agreement |

ICC = intraclass correlation coefficient. Pre-study validation conducted with 10 non-participant junior ophthalmologists.

**Supplementary Table S4.** Results of Statistical Assumption Verification

**Normality Testing (Shapiro–Wilk Test, α = 0.05)**

| **Variable** | **AI-Integrated Cohort** | **Conventional Cohort** | **Distribution** |
| --- | --- | --- | --- |
| Diagnostic confidence score (T0) | W = 0.971, p = 0.243 | W = 0.968, p = 0.198 | Normal |
| Diagnostic confidence score (T1) | W = 0.965, p = 0.152 | W = 0.972, p = 0.261 | Normal |
| Strain adjustment ability (T0) | W = 0.974, p = 0.312 | W = 0.969, p = 0.214 | Normal |
| Strain adjustment ability (T1) | W = 0.970, p = 0.228 | W = 0.966, p = 0.168 | Normal |
| Treatment-plan adaptation (T0) | W = 0.976, p = 0.358 | W = 0.973, p = 0.289 | Normal |
| Treatment-plan adaptation (T1) | W = 0.968, p = 0.198 | W = 0.971, p = 0.241 | Normal |
| Scheme-decision Kappa (T0) | W = 0.931, p = 0.006 | W = 0.928, p = 0.004 | Non-normal |
| Scheme-decision Kappa (T1) | W = 0.924, p = 0.003 | W = 0.936, p = 0.010 | Non-normal |
| Decision-point concordance (T0) | W = 0.938, p = 0.012 | W = 0.941, p = 0.015 | Non-normal |
| Decision-point concordance (T1) | W = 0.927, p = 0.004 | W = 0.933, p = 0.008 | Non-normal |
| Case-analysis time (T0) | W = 0.942, p = 0.016 | W = 0.939, p = 0.013 | Non-normal |
| Case-analysis time (T1) | W = 0.935, p = 0.009 | W = 0.944, p = 0.019 | Non-normal |
| Emergency response time (T0) | W = 0.946, p = 0.022 | W = 0.943, p = 0.018 | Non-normal |
| Emergency response time (T1) | W = 0.937, p = 0.011 | W = 0.940, p = 0.014 | Non-normal |

Normally distributed variables were analyzed using parametric tests (paired t-test, independent t-test); non-normally distributed variables were analyzed using nonparametric tests (Wilcoxon signed-rank, Mann–Whitney U).

**Variance Homogeneity (Levene's Test, α = 0.05)**

| **Variable (T1)** | **F** | **p** | **Interpretation** |
| --- | --- | --- | --- |
| Diagnostic confidence score | F = 2.31 | 0.132 | Homogeneous |
| Strain adjustment ability | F = 1.87 | 0.175 | Homogeneous |
| Treatment-plan adaptation | F = 1.54 | 0.217 | Homogeneous |

All normally distributed continuous outcomes met the assumption of variance homogeneity, supporting the use of standard independent-samples t-tests without Welch's correction.

**Sphericity Testing for Repeated-Measures ANOVA (Mauchly's Test)**

| **Outcome (T0–T1–T2)** | **Mauchly's W** | **p** | **Correction Applied** |
| --- | --- | --- | --- |
| Cross-center consultation Kappa | 0.891 | 0.038 | Greenhouse–Geisser (ε = 0.912) |
| Teaching-radiation index | 0.923 | 0.087 | None (sphericity met) |
| Follow-up management accuracy | 0.876 | 0.024 | Greenhouse–Geisser (ε = 0.898) |

Where sphericity was violated, Greenhouse–Geisser correction was applied. Time × group interaction effects were significant for all sustainability outcomes (all p < 0.001), confirming differential temporal trajectories between cohorts.

**Supplementary Table S5. Balance of Clinical Exposure Variables Between Cohorts During the Intervention Period**

Clinical exposure was tracked prospectively using institutional duty rosters and biweekly participant self-reports. All variables refer to the mean value observed across the combined 12-week intervention and 4-week follow-up period.

| **Variable** | **AI-Integrated Cohort (n = 50)** | **Conventional Cohort (n = 50)** | **Test Statistic** | **p** |
| --- | --- | --- | --- | --- |
| Weekly clinical workload (hours), mean ± SD | 42.6 ± 5.8 | 42.1 ± 6.1 | t = 0.42, df = 98 | 0.671 |
| Subspecialty rotation—retina (n, %) | 12 (24.0) | 13 (26.0) | χ² = 0.05, df = 1 | 0.822 |
| Subspecialty rotation—cornea/anterior segment (n, %) | 11 (22.0) | 10 (20.0) | χ² = 0.06, df = 1 | 0.808 |
| Subspecialty rotation—glaucoma (n, %) | 10 (20.0) | 12 (24.0) | χ² = 0.24, df = 1 | 0.628 |
| Subspecialty rotation—pediatric/strabismus (n, %) | 9 (18.0) | 8 (16.0) | χ² = 0.07, df = 1 | 0.789 |
| Subspecialty rotation—general (n, %) | 8 (16.0) | 7 (14.0) | χ² = 0.08, df = 1 | 0.782 |
| Weekly outpatient clinic sessions, mean ± SD | 3.8 ± 0.9 | 3.7 ± 1.0 | t = 0.53, df = 98 | 0.599 |
| Weekly inpatient ward duty (hours), mean ± SD | 14.2 ± 3.1 | 14.0 ± 3.3 | t = 0.31, df = 98 | 0.756 |
| Weekly on-call emergency coverage (hours), mean ± SD | 8.4 ± 2.7 | 8.7 ± 2.5 | t = 0.58, df = 98 | 0.566 |
| Assisted surgical participation (cases/week), mean ± SD | 2.9 ± 1.1 | 2.8 ± 1.2 | t = 0.43, df = 98 | 0.666 |
| Real-world DR cases encountered (per participant, total) | 27.4 ± 6.9 | 26.8 ± 7.2 | t = 0.43, df = 98 | 0.670 |
| Working independently without direct supervision (n, %) | 0 (0.0) | 0 (0.0) | — | — |

SD = standard deviation. All participants worked under the direct supervision of attending or associate chief physicians; none held independent operative privileges during the study period. Between-group comparisons used independent-samples t-tests for continuous variables and χ² tests for categorical variables. No variable reached statistical significance, indicating balanced clinical exposure between the two cohorts.

**Supplementary Table S6. Self-Directed Learning and External Educational Activities During the Study Period**

Participants completed biweekly self-report logs capturing time devoted to self-directed learning outside of curricular hours, informal consultation with supervising attendings on DR-related cases, and participation in external continuing medical education activities.

| **Activity** | **AI-Integrated Cohort (n = 50)** | **Conventional Cohort (n = 50)** | **Test Statistic** | **p** |
| --- | --- | --- | --- | --- |
| Self-directed DR reading (hours/week), mean ± SD | 3.8 ± 1.2 | 3.6 ± 1.3 | t = 0.80, df = 98 | 0.425 |
| Informal consultation with attending on DR (occasions/week), mean ± SD | 2.1 ± 0.9 | 2.0 ± 0.8 | t = 0.59, df = 98 | 0.559 |
| External CME participation (hours over 16 weeks), mean ± SD | 6.4 ± 2.3 | 6.1 ± 2.5 | t = 0.63, df = 98 | 0.532 |
| Preparation for professional advancement exam (yes, n, %) | 8 (16.0) | 9 (18.0) | χ² = 0.07, df = 1 | 0.789 |
| Use of external AI diagnostic tools outside curriculum (yes, n, %) | 0 (0.0) | 0 (0.0) | — | — |

CME = continuing medical education; SD = standard deviation. Between-group differences were not statistically significant for any activity (all p > 0.05), supporting the conclusion that observed outcome differences are unlikely to be attributable to differential self-directed learning or external educational activities. Access to the AI simulation platform was gated by individual credentials with audited usage logs; no participant in either cohort accessed the platform outside the pre-specified curricular hours.

**Supplementary Table S7. Time × Group Interaction Effects from Repeated-Measures ANOVA**

Planned repeated-measures analyses of variance were conducted for each primary and secondary outcome across the three assessment time points (T0, T1, and, where applicable, T2). The Time × Group interaction term tests whether the pattern of change over time differs between the AI-integrated and conventional cohorts. Greenhouse–Geisser correction was applied where Mauchly's test indicated a violation of the sphericity assumption. Partial η² is reported as the effect-size estimate for each interaction term.

| **Outcome** | **F** | **df** | **p (uncorrected)** | **p (GG-corrected)** | **Partial η²** |
| --- | --- | --- | --- | --- | --- |
| Scheme-decision Kappa (T0–T1–T2) | 384.6 | 2, 196 | <0.001 | <0.001 | 0.71 |
| Key decision-point concordance (T0–T1–T2) | 342.1 | 2, 196 | <0.001 | <0.001 | 0.68 |
| Differential diagnosis coverage (T0–T1–T2) | 315.7 | 2, 196 | <0.001 | <0.001 | 0.65 |
| Diagnostic confidence score (T0–T1) | 298.4 | 1, 98 | <0.001 | N/A | 0.63 |
| Decision uncertainty score (T0–T1) | 245.8 | 1, 98 | <0.001 | N/A | 0.58 |
| Strain adjustment ability (T0–T1) | 312.6 | 1, 98 | <0.001 | N/A | 0.66 |
| Complex-case diagnostic accuracy (T0–T1) | 276.2 | 1, 98 | <0.001 | N/A | 0.62 |
| Mean case-analysis time (T0–T1) | 231.4 | 1, 98 | <0.001 | N/A | 0.56 |
| Prognostic concordance (RMSE) (T0–T1) | 204.7 | 1, 98 | <0.001 | N/A | 0.51 |
| Acute-complication response time (T0–T1) | 219.8 | 1, 98 | <0.001 | N/A | 0.55 |
| Comorbidity recognition accuracy (T0–T1) | 263.5 | 1, 98 | <0.001 | N/A | 0.60 |
| High-risk-factor discrimination (AUC) (T0–T1) | 187.9 | 1, 98 | <0.001 | N/A | 0.42 |
| Individualized treatment-plan adaptation (T0–T1) | 351.9 | 1, 98 | <0.001 | N/A | 0.69 |
| Cross-center consultation Kappa (T0–T1–T2) | 267.3 | 2, 196 | <0.001 | <0.001 (ε=0.912) | 0.60 |
| Homogenization teaching-radiation index (T0–T1–T2) | 312.8 | 2, 196 | <0.001 | <0.001 | 0.65 |
| Long-term follow-up management accuracy (T0–T1–T2) | 289.5 | 2, 196 | <0.001 | <0.001 (ε=0.898) | 0.62 |

F = ANOVA F-statistic for Time × Group interaction; df = degrees of freedom (numerator, denominator); GG = Greenhouse–Geisser; ε = sphericity correction epsilon. All interaction terms reached statistical significance at p < 0.001, indicating that the temporal trajectory of improvement differed significantly between the two cohorts across every outcome examined.

**Supplementary Note S1. Detailed Exposition of the Chinese Medical Professional Title System**

The Chinese medical professional title system comprises four hierarchical tiers—resident physician (*zhùyuàn yīshī*), attending physician (*zhǔzhì yīshī*), associate chief physician (*fù zhǔrèn yīshī*), and chief physician (*zhǔrèn yīshī*)—which are determined primarily by years of post-licensure service and national qualification examination performance rather than by clinical training stage, and do not correspond directly to Western residency or fellowship designations [20,21]. Ranking within this system is linked not only to basic salary but also to institutional prestige; however, the evaluation criteria for title promotion—which emphasize publications and examination performance—do not necessarily reflect clinical capability, a distinction that is critical for interpreting the participant characteristics reported herein [22]. The 2021 Medical Practitioners Law of the People's Republic of China further codifies this administrative classification as being distinct from clinical competency certification [23]. In the present study, all participants met the pre-specified inclusion criterion of 1–5 years of clinical experience; the title distribution reported in Table 1 therefore reflects the administrative classification system rather than functional seniority equivalent to Western training-stage designations, and is provided solely for demographic transparency.

**Supplementary Note S2. Worked Example of the Per-Participant Key Decision-Point Concordance and Differential Diagnosis Coverage Calculations**

To illustrate how the per-participant summary statistics reported in Table 2 of the main manuscript are derived from the raw scoring sheets, we present a worked example for a representative participant assigned to the AI-integrated cohort at the immediate post-intervention time point (T1). The example is constructed from the actual raw scoring data of participant AI-27, whose per-participant values fall very close to the reported AI-integrated cohort medians and therefore serve as a transparent, non-idealized reference.

**Key decision-point concordance.** For each of the 10 standardized assessment cases, participant AI-27 received a graded score of 0.0, 0.5, or 1.0 at each of the five pre-specified decision points (lesion-staging determination, macular edema assessment, initial treatment modality selection, follow-up frequency, and acute-complication early-warning activation). The raw per-decision-point scores across the 10 cases yielded per-case sums (out of 5) of, respectively, 5.0, 5.0, 4.5, 5.0, 4.5, 5.0, 5.0, 4.5, 5.0, and 5.0. Converting each per-case sum to a percentage by dividing by 5 and multiplying by 100 produces per-case concordance percentages of 100%, 100%, 90%, 100%, 90%, 100%, 100%, 90%, 100%, and 100%. The arithmetic mean of these ten per-case percentages is (100 + 100 + 90 + 100 + 90 + 100 + 100 + 90 + 100 + 100) / 10 = 970 / 10 = 97.0%. This 97.0% value is the per-participant key decision-point concordance score entered into the cohort-level analysis for participant AI-27.

Aggregation to the reported median of 95.5%. Once every participant's per-participant percentage was computed in this manner, the 50 values in the AI-integrated cohort were sorted in ascending order and the median was calculated using the R Type 7 quantile algorithm (identical to the SPSS default), which linearly interpolates between neighboring ordered values. For the AI-integrated cohort at T1, the 25th ordered value was 95.0% and the 26th ordered value was 96.0%, so the median resolves to (95.0 + 96.0) / 2 = 95.5%. The first quartile (Q1) is located at position 1 + (50 − 1) × 0.25 = 13.25 of the sorted sequence; the 13th ordered value was 93.0% and the 14th was 96.0%, and linear interpolation yields Q1 = 93.0 + 0.25 × (96.0 − 93.0) = 93.75%, which is reported to one decimal place as 93.8%. The third quartile (Q3) is located at position 1 + (50 − 1) × 0.75 = 37.75; the 37th and 38th ordered values were 97.0% and 98.0%, and linear interpolation yields Q3 = 97.0 + 0.75 × (98.0 − 97.0) = 97.75%, which is reported as 97.8% after the standard one-decimal rounding rule applied consistently across the manuscript. This reconstruction illustrates why summary statistics such as 95.5% (IQR: 93.8–97.8%) can arise even though any single case can only contribute concordance values in 10-percentage-point increments and any single participant's per-participant score lies on a 1-percentage-point grid, and confirms that the reported values are internally consistent with the scoring rubric described in Section 2.6.1(b).

**Differential diagnosis coverage.** An analogous calculation applies to the differential diagnosis coverage metric described in Section 2.6.1(c). For participant AI-27, the graded scoring (0.0, 0.5, or 1.0 per differential, applied across the eight pre-specified entities per case) produced per-case coverage sums (out of 8) of, respectively, 8.0, 8.0, 7.5, 8.0, 7.5, 8.0, 8.0, 7.5, 8.0, and 8.0. Dividing each per-case sum by 8 and expressing as a percentage yields per-case coverage values of 100%, 100%, 93.75%, 100%, 93.75%, 100%, 100%, 93.75%, 100%, and 100%. The arithmetic mean across the ten cases is (100 + 100 + 93.75 + 100 + 93.75 + 100 + 100 + 93.75 + 100 + 100) / 10 = 981.25 / 10 = 98.125%, which is entered as the participant's coverage score and contributes to the cohort median of 97.0% (IQR 95.5–98.2%) reported in Table 2 following the same R Type 7 quantile interpolation.

Independent re-verification. Following the associate editor's request, all per-participant values contributing to the primary outcome summary statistics reported in Table 2 were independently recomputed from the raw scoring sheets by two members of the investigator team working in parallel, with 100% agreement on the recomputed values. The recomputed cohort-level medians and interquartile ranges matched the values previously reported to the first decimal place, and no revision to the numerical results in the main text was required.
